# The Best Tooth Wash

**Published:** 1876-02

**Authors:** 


*The Best Tooth Wash,*

because the safest, most familiar, and most universally accessible, and most invariably applicable and efficient, where specific dental science is not sought, is a piece of common white soap with a brush of moderate stiffness. The correspondent of a medical cotemporary inquires as to the truth of the statement, to which the editor replies simply, "*It is nonsense!*" What are the ascertained facts of the case? "Tartar on the teeth," is a familiar expression. Microscopical examination shows that millions of living things are there,—that there are mainly two kinds, and that the larger class are instantaneously killed by soap-suds, when strong acids have no effect whatever. Here is a simple fact, on which eminent dentists have based the practical advice to use common white soap as a corrector and preventive of tartar on the teeth to a considerable extent, so that we almost look contemptuously on the flippancy of an editor who would write "nonsense" to such a statement, when most probably he never made a microscopical examination of this "tartar," and never performed an experiment on the living things which seem to form it from a product furnished by the animal economy, very much perhaps as coral is formed by myriads of invisible creatures from material found in the waters of the sea.

